# Alternative Isoforms as Molecular Modulators of Cellular Senescence in Cancer

**DOI:** 10.3390/cancers18203301

**Published:** 2026-10-13

**Authors:** José Manuel García-Heredia

**Affiliations:** 1 Departamento de Bioquímica Vegetal y Biología Molecular, Universidad de Sevilla, 41012 Sevilla, Spain; jmgheredia@us.es; 2 Instituto de Biomedicina de Sevilla (IBIS), Hospital Universitario Virgen del Rocío (HUVR), Consejo Superior de Investigaciones Científicas (CSIC), Universidad de Sevilla, 41013 Sevilla, Spain; 3 CIBER de Cáncer (CIBERONC), Instituto de Salud Carlos III, 28029 Madrid, Spain

**Keywords:** cellular senescence, alternative splicing, protein isoforms, SASP, cell plasticity, tumor suppression, therapy resistance, tumor microenvironment

## Abstract

Cellular senescence is a stress-induced, stable proliferative arrest that acts as a potent barrier to tumorigenesis, yet the senescence-associated secretory phenotype that accompanies it can paradoxically fuel inflammation, angiogenesis and the phenotypic plasticity contributing to tumor heterogeneity, therapy resistance and cancer stem cell emergence. A largely unexplored molecular layer in the senescence program is alternative pre-mRNA splicing, which generates protein isoforms with functionally divergent effects on cell-cycle arrest, apoptosis and the secretory program. More than 90% of human multi-exon genes are alternatively spliced, so most of the nearly 1000 genes linked to senescence are expected to produce several isoforms, yet fewer than twenty have been examined at this level, and direct evidence is available for only about half of these. We review how alternative splicing, together with related mechanisms such as alternative promoter usage and alternative reading-frame selection, modulates distinct outputs of the senescence program, including commitment, persistence, escape and SASP composition, across gene groups such as the TP53/TP63/TP73 family, MDM2/MDM4 and the androgen receptor locus. This evidence ranges from direct causal manipulation to correlative or mechanistically adjacent findings, classified explicitly by evidence level. Direct causal evidence remains limited, making isoform-resolved functional validation a major unmet need in the field.

## Simple Summary

Cells exposed to damaging or stressful conditions can enter senescence, a stable state in which they stop dividing and undergo functional changes, including the production of secreted factors known as the senescence-associated secretory phenotype. Senescence can protect against tumor formation by preventing damaged cells from continuing to proliferate, but persistent senescent cells can also promote tumor progression in some contexts. Part of this variability may arise because cells can splice or start reading their genetic instructions differently, producing several versions, or isoforms, of the same protein. Most human genes produce such versions; of the nearly one thousand genes linked to senescence, only a handful have been studied. This review brings together how these versions influence whether cells enter, maintain, or escape senescence. Organizing the evidence by how directly each finding has been demonstrated clarifies what is established, what is speculative, and where the most promising research opportunities lie.

## 1. Introduction

### 1.1. Cellular Senescence: Tumor-Suppressive Arrest, Tumor-Permissive Secretion

Cellular senescence is a durable proliferative arrest that cells adopt in response to a wide range of stresses, including replicative exhaustion from progressive telomere shortening, oncogene activation, DNA damage, mitochondrial dysfunction and chemotherapeutic insult [1,2,3]. It is generally irreversible, although cell-cycle re-entry occurs, particularly in tumor cells [3]. Senescent cells remain metabolically active and undergo a characteristic set of morphological and biochemical changes: increased cell size, chromatin reorganization, and elevated senescence-associated β-galactosidase (SA-β-gal) activity. They also acquire the senescence-associated secretory phenotype (SASP), a program of cytokines, chemokines, growth factors and matrix-remodeling proteases that allows senescent cells to signal to their microenvironment [4,5,6]. Senescence is one of the principal tumor-suppressive mechanisms, alongside apoptosis; oncogenic RAS triggers a protective, p53- and p16INK4a-dependent senescence response rather than transformation [7]. Senescence is not a single state but a family of related ones, and a mechanism established in one of them does not automatically apply to the rest. Robust identification of senescence requires more than one independent marker, such as SA-β-gal positivity together with stable cell-cycle-inhibitor induction or characteristic chromatin changes [3,8].

This protective role, however, is only one side of a duality inherent to the senescence program. The same secreted factors that recruit immune effectors to clear pre-malignant senescent cells can instead generate a pro-inflammatory, pro-angiogenic microenvironment when senescent cells persist chronically or accumulate with age [3,9]. That microenvironment favors the proliferation of neighboring damaged or transformed cells, promotes epithelial–mesenchymal transition (EMT)-like changes, and can even support the outgrowth of the very tumors that senescence was meant to suppress [4,10]. This duality is well established for the senescence program as a whole; how isoform diversity contributes to it at the level of individual genes is far less systematically understood.

### 1.2. Cell Plasticity and Its Intersection with Senescence in Tumor Progression

In parallel to senescence, tumor cells display a remarkable capacity for phenotypic plasticity: the ability to transition between differentiation states, to dedifferentiate toward stem-like phenotypes, and to interconvert between epithelial and mesenchymal-like identities in response to microenvironmental cues [11,12]. This plasticity can generate cancer stem cells from more differentiated tumor cells; contributes to intratumoral heterogeneity and resistance to chemotherapy, radiotherapy and targeted agents; and is implicated in metastasis, recurrence and minimal residual disease [12,13]. Senescence and plasticity are not independent: cells that escape senescence resume proliferation with a more plastic, stem-like identity than the one they had before arrest [14].

### 1.3. Alternative Splicing as an Underexplored Layer of the Senescence Program

Alternative pre-mRNA splicing can switch a single gene between tumor-suppressive and tumor-promoting outputs, placing it at the intersection of senescence and plasticity. As to whether individual isoform switches operate, there is, at present, a proposal: no isoform switch has been followed from a senescence outcome to a plasticity outcome within a single experimental system. Splicing is carried out by the spliceosome, which removes introns from precursor mRNAs and can join exons in alternative combinations, generating multiple transcripts, and therefore multiple protein isoforms, from a single gene [15,16,17]. It affects more than 90% of human multi-exon genes and is a fundamental source of proteomic diversity beyond what is encoded in the genome [12,16,18,19]. Splicing factors are frequently overexpressed, mutated or otherwise dysregulated in cancer, and this dysregulation reshapes the isoform repertoire of oncogenes, tumor suppressors and apoptotic regulators, contributing to essentially every cancer hallmark [12,20]. This dysregulation can in principle be exploited therapeutically, from antisense splice-switching oligonucleotides to small-molecule spliceosome modulators [21].

Even so, the question of how splicing-generated isoforms influence entry into, maintenance of or escape from senescence has been addressed gene by gene rather than synthesized. A small number of studies have begun to make this connection explicit: an oncogenic splicing factor can trigger a senescence checkpoint without acting through its splicing targets [22]. Global spliceosome dysfunction can act as a senescence trigger by shifting the isoform balance of individual senescence-regulatory genes such as MDM4 [23]. A dedicated siRNA screen has further shown that PTBP1 is required for the pro-tumorigenic inflammatory secretome of oncogene-induced senescent (OIS) cells [24]. At the level of individual isoforms, this question has received far less attention than the contribution of alternative splicing to the hallmarks of cancer, to senescence and organismal aging, or to cancer stem-cell plasticity [20,25,26].

Two curated gene sets annotate 968 human genes as senescence-associated [6,27]. Since most human multi-exon genes are alternatively spliced, most of these genes are expected to produce more than one transcript and protein isoform, and for many of them individual isoforms have been characterized in other biological contexts [28,29,30,31,32,33]. For most of them, whether the isoform balance affects entry into, persistence in or escape from senescence, or the composition of the SASP, has not been examined.

## 2. Molecular Mechanisms Generating Senescence-Relevant Isoforms

### 2.1. Isoform-Generating Mechanisms Among Senescence-Associated Genes

Isoform pairs relevant to senescence can arise through several recurring, non-mutually-exclusive mechanisms. Exon skipping removes one or more internal exons from the mature transcript, as in MCL1, where it converts the anti-apoptotic MCL-1L into the pro-apoptotic MCL-1S [34,35]. Intron retention preserves part of an intron within the mature mRNA and often introduces a premature stop codon: in TP53, retention of intron 2 forces translational reinitiation downstream, generating Δ40p53 [36]. Alternative 5′ or 3′ splice-site selection produces smaller differences between isoforms, as in BCL2L1, where use of a distal 5′ splice site within exon 2 generates the short, pro-apoptotic Bcl-xS instead of the anti-apoptotic Bcl-xL [37]. Isoform diversity can also arise before splicing, through alternative promoter or transcription-start-site usage, which generates proteins with distinct N-termini but a shared C-terminal region. This mechanism produces Δ133p53, ΔNp63 and ΔNp73, which lack all or part of the N-terminal transactivation domain of the full-length TA isoforms [38,39,40,41,42]. Finally, a cassette exon carrying an in-frame premature stop codon can redirect translation, as in TP53BP2. Inclusion of exon 3 interrupts the open reading frame initiated at the canonical AUG, forcing translational reinitiation at an internal AUG in exon 6 and generating the short isoform 53BP2S [43]. In Δ40p53 and 53BP2S, the splicing event acts through alternative translation initiation, which determines the final protein product.

### 2.2. The Spliceosome and Individual Splicing Factors as Senescence Checkpoints

Beyond the isoforms themselves, the splicing machinery is subject to senescence-associated regulation, and this regulation is causally linked to senescence commitment. Across replicative, stress-induced and telomere-uncapping-induced senescence models, core spliceosome components and splicing regulators were generally reduced. Pharmacological inhibition of splicing with herboxidiene or pladienolide B, or siRNA-mediated depletion of individual spliceosome components, was sufficient to induce senescence in normal and cancer cell lines, indicating that impaired splicing acts as a trigger rather than a consequence of senescence [23]. Across the same senescence models, MDM4 splicing converged on a transition from the full-length MDM4-FL transcript to the truncated MDM4-S variant; depletion of MDM4-FL alone was sufficient to induce senescence markers, whereas MDM4-S did not drive senescence onset [23].

Individual splicing factors act as senescence checkpoints as well: overexpression of SRSF1 triggers a form of OIS through the ribosomal-stress-response pathway. SRSF1 binds to the ribosomal protein RPL5 and to MDM2, sequestering MDM2 away from p53 and stabilizing p53, so that an oncogenic splicing factor curtails its own transforming potential through a senescence checkpoint [44]. Downregulation of SRSF3, which occurs physiologically during senescence, releases its repression of TP53 pre-mRNA splicing toward the p53β isoform and promotes senescence [45]. SRSF7 downregulation likewise induces senescence, in this case through generation of MDM2 splice variants [46]. All three converge on the p53–MDM2 axis, which thus appears to be the main point at which changes in splicing-factor abundance are translated into a senescence decision. A different output is controlled by PTBP1, which regulates the alternative splicing of genes involved in intracellular trafficking, including EXOC7; its depletion selectively attenuates the pro-inflammatory, pro-tumorigenic secretory phenotype without reversing the senescent arrest [24].

Splicing outcome is also shaped upstream of individual factors, by the three-dimensional organization of the genome. Genes located toward the nuclear periphery are spliced by exon definition and alternatively spliced mainly through exon skipping. By contrast, genes located toward the nuclear center are spliced by intron definition and are prone to intron retention, and shifting a gene’s nuclear position changes its splicing outcome [47]. In senescent cells, CTCF is reorganized into senescence-induced clusters assembled on nuclear speckles, and this clustering instructs the senescence-associated alternative splicing program: its disruption almost fully reverts splicing patterns and delays senescence onset [48]. Splicing control in senescence therefore operates at three levels: the integrity of the spliceosome, individual splicing factors and, upstream of both, nuclear architecture.

## 3. Study Selection, Evidence Grading and Senescence Context

### 3.1. Literature Search and Evidence-Level Framework

The starting point was the union of two curated senescence gene sets, CellAge build 3 (866 genes) [27] and SenMayo (125 genes) [6], comprising 968 unique genes. For each gene, PubMed was searched in August and September 2026 for protein isoforms and for evidence relating any of them to cellular senescence. The core set of studies thus assembled was expanded by citation chaining, following the reference lists and related-article suggestions of the retrieved papers, which also brought in genes absent from both gene sets (TP73, MDM4 and EXOC7). References on senescence biology, splicing mechanisms and clinical context were added where needed to support the discussion. An isoform was retained when it arises from alternative splicing, alternative promoter usage or alternative translation initiation and has a documented or biologically plausible relationship to senescence; the strength of that relationship is what the evidence level captures. Evidence levels were assigned on the basis of the primary study, not of its description in secondary sources.

Because the strength of evidence linking a given isoform to cellular senescence varies substantially across genes, each isoform pair was classified into one of four evidence levels, ranked from strongest to weakest. The level grades the switch between the isoforms of a pair, not the senescence activity of either product considered alone. Manipulating one member of a pair counts as direct evidence when it changes the ratio between the isoforms, and not when the two products arise independently and the level of one does not affect the other. The framework follows the general logic of graded evidence-strength schemes used in genomics and precision oncology [49,50], without adopting any single existing classification. Direct evidence (L1) means that experimental manipulation of the isoform itself, such as knockdown, overexpression, splice-switching, or an equivalent molecular perturbation, directly altered a senescence phenotype. Senescence-associated evidence (L2) means that the isoform has been linked to a senescence phenotype without direct experimental manipulation demonstrating that the isoform itself is responsible for the phenotype. Mechanistically adjacent evidence (L3) means that the switch between the isoforms has not been tested in senescence: the role of one product, or of the total protein, may be documented, but the contribution of the isoform balance is inferred. Unresolved (L4) means that a senescence link is speculative or has not been studied at all. The level assigned to each isoform is given at its first mention, and causal language is reserved for relationships with direct evidence; the other three levels are described in associative terms.

Each isoform also reflects one of four isoform-generating mechanisms: alternative splicing proper (exon skipping, intron retention, or alternative 5′ or 3′ splice-site selection); alternative promoter or transcription-start-site usage; alternative promoter usage coupled to an alternative reading frame on a downstream shared exon; and alternative translation initiation coupled to a splicing event. These processes are mechanistically distinct and should not be conflated under a single “splicing” label. Each isoform is also assigned to the functional axis of the senescence program it most directly affects: entry/commitment, escape, persistence/survival, or SASP regulation. Isoform-level evidence in relation to senescence was identified for 19 genes, 9 of them with direct evidence (Table 1).

### 3.2. Senescence Models and the Interpretation of Isoform-Specific Mechanisms

Isoform–senescence relationships have not been established under a single senescence program. They span replicative, oncogene-induced and therapy-induced senescence, stress-induced states such as tetraploidization, and senescence triggered by oncogene withdrawal or by loss of a tumor suppressor.

In normal human fibroblasts, induction of p53β with diminished Δ133p53 is specific to replicative senescence and is not observed in OIS [39]. The MDM4-FL to MDM4-S shift, by contrast, is common to replicative senescence, oxidative stress and telomere uncapping [23]. Whether an isoform switch marks senescence in general or one route into it is therefore an empirical question, and for most of these genes the switch has been examined under a single trigger (Table 2).

## 4. The TP53 Family: A Paradigm of Isoform-Driven Senescence Regulation

The p53 family (TP53, TP63, TP73) provides a well-documented example of a gene family in which alternative splicing and alternative promoter usage generate isoforms with directly antagonistic effects on senescence. It is also the group of genes with the strongest experimental evidence for a differential role of isoforms in senescence.

### 4.1. TP53 Isoforms: Δ133p53, p53β and Δ40p53

The human TP53 gene encodes twelve reported protein isoforms, and this diversity has been proposed to explain much of the pleiotropy of p53 function in senescence, aging and cancer [42,86]. Three of them have been related to senescence: Δ133p53 and p53β (L1) as well as Δ40p53 (L1, context-dependent) (Figure 1A). Δ133p53 (and its α, β and γ carboxy-terminal variants) lacks the first 132 amino acids of full-length p53, including both transactivation domains and the start of the DNA-binding domain, and behaves as an endogenous inhibitor of full-length p53 activity. By contrast, p53β retains the N-terminal transactivation domain but has an altered C-terminus caused by intron 9 splicing that removes the oligomerization domain [39,42]. In normal human fibroblasts, induction of p53β together with diminished Δ133p53 accompanies replicative, but not oncogene-induced, senescence. Knockdown of endogenous Δ133p53 by siRNA is sufficient to induce senescence via regulation of p21WAF1 and other p53 target genes, and, in overexpression experiments, p53β cooperates with full-length p53 to promote senescence while Δ133p53 antagonizes it [39]. In peripheral blood CD8^+^ T lymphocytes, senescent cells accumulate with age and show decreased Δ133p53 and increased p53β expression in vivo, a signature also found in human lung tumor-infiltrating CD8^+^ T lymphocytes. Reconstituting Δ133p53 or the co-stimulatory receptor CD28 in poorly proliferative Δ133p53-low CD8^+^CD28^−^ cells restored proliferation, extended replicative lifespan and rescued senescence phenotypes. Conversely, Δ133p53 knockdown or p53β overexpression in CD8^+^CD28^+^ cells was sufficient to inhibit proliferation and induce senescence, establishing both isoforms as physiological regulators of replicative senescence in vivo [41]. The balance between the two isoforms is set at two levels: Δ133p53 is cleared by autophagy during replicative senescence [87], and SRSF3 downregulation during senescence favors p53β [45].

Δ40p53 (also known as p53/p47, and as ΔN-p53 in [52]) arises by alternative translation initiation at AUG40 and by retention of intron 2, whose in-frame stop codons block translation from AUG1. The protein retains the DNA-binding and oligomerization domains but lacks the first transactivation domain [36,52]. The direction of its effect on senescence is cell-type dependent [88]. In p53-null human lung cancer cells, Δ40p53 did not suppress growth by itself but blocked the suppression by co-expressed full-length p53, and in mouse fibroblasts it repressed the transactivation driven by the full-length protein [52]. In hepatocellular carcinoma cells expressing Δ40p53 from a targeted endogenous allele, TP53 knockdown attenuated SA-β-gal positivity and p21 induction. Ectopic Δ40p53 restored both markers and also suppressed proliferation, independently of TP53 mutation status [51].

### 4.2. TP63 and TP73 Isoforms: Opposing Effects on Senescence and Immortalization

The TP63 gene similarly encodes two isoform classes with opposing functions, TAp63 and ΔNp63 (L1): TAp63 isoforms retain the p53-like N-terminal transactivation domain and can induce apoptosis and cell-cycle arrest. ΔNp63 isoforms, generated from an internal promoter (Figure 1A), lack that domain and instead enhance proliferation and inhibit apoptosis, an opposition analogous to that of Δ133p53/p53β [40]. In a Tet-on inducible mouse system, TAp63 induced a bona fide senescence program and suppressed tumorigenesis, establishing it as an in vivo tumor suppressor acting at least in part through senescence rather than apoptosis or cell-cycle arrest [40].

TP73 has a similar isoform architecture (Figure 1A): ΔNp73 (L1) acts as a potent transdominant inhibitor of both wild-type p53 and TAp73, counteracting their transactivation, apoptotic and growth-suppressive functions. This N-terminally truncated isoform, generated from an internal promoter, is frequently overexpressed in human tumors [38]. In primary mouse embryonic fibroblasts (MEFs), ΔNp73 confers resistance to replicative senescence and immortalizes them at a roughly 1000-fold higher frequency than occurs spontaneously, cooperating with c-Myc and E1A to promote proliferation and colony formation while compromising p53-dependent apoptosis. ΔNp73 rescues cells from oncogenic RAS-induced senescence and cooperates with oncogenic K-RAS, N-RAS and H-RAS in vivo to produce tumors in mice [38].

### 4.3. A Shared Architecture Across the p53 Family

The TP53, TP63 and TP73 data illustrate a recurring architectural principle: alternative promoter usage and splicing generate isoforms with different transactivation capacities and, consequently, distinct effects on senescence and tumor suppression. Across these loci, TA or full-length isoforms can promote senescence, whereas ΔN isoforms can antagonize p53-family tumor-suppressive functions, although the mechanisms and strength of evidence differ between family members. This generalization does not hold uniformly even within TP53: Δ40p53, which also lacks the first transactivation domain, shows cell-type-dependent effects that in some contexts oppose the ΔN pattern. The balance between these isoforms, rather than the presence or absence of the gene, can therefore influence whether the pathway promotes senescence or permits continued, potentially malignant, proliferation.

## 5. The p53 Regulatory Network: Splicing as a Senescence Switch

Because MDM2 and MDM4 are the principal negative regulators of p53 [89,90], any mechanism that alters their isoform balance has an immediate route to the p53-dependent senescence program. In normal and cancer cell lines, replicative senescence, oxidative stress and telomere uncapping all converged on a shift from full-length MDM4 (MDM4-FL, a potent p53 inhibitor) to a C-terminally truncated MDM4-S variant (L1). This isoform is generated by skipping of exon 6, which shifts the reading frame and introduces a premature termination codon (Figure 1A). This FL-to-S shift accompanied senescence commitment, and the functional evidence for its contribution rests on the loss of MDM4-FL rather than on the gain of MDM4-S. Knockdown of endogenous MDM4 induced senescence, and recombinant MDM4-S did not prevent it; an MDM4-FL-specific shRNA increased SA-β-gal activity, slowed proliferation and significantly upregulated p21, CCND1 and CCND2. By contrast, MDM4-S overexpression did not affect the onset of senescence and improved senescent-cell survival. The shift was also reproduced, without any senescence-inducing stimulus, by pharmacological or genetic impairment of spliceosome activity [23]. Structurally, MDM4-S retains the p53-binding domain but lacks the C-terminal RING domain required for heterodimerization with MDM2, which would be expected to relieve MDM2-mediated repression of p53. However, MDM4-S does not inhibit p53 transactivation, and splicing-defect-induced senescence occurs largely independently of p53, leaving the link between the FL-to-S shift and p53 activity unresolved [23].

The MDM2 gene itself generates numerous alternatively spliced transcripts in tumor and normal tissue, but the functional characterization of individual variants in the context of senescence remains limited. MDM2-A, MDM2-B and MDM2-C (L1, context-dependent) are three of the most common, and all lack the N-terminal p53-binding domain but retain the C-terminal RING domain and remain able to bind MDM2-FL (Figure 1A). When overexpressed in MCF-7 breast cancer cells in the absence of chemotherapy, MDM2-B and MDM2-C significantly increased the fraction of senescent cells, while MDM2-A produced a smaller, non-significant increase. This effect was lost in doxorubicin-treated cells, where MDM2-B and MDM2-C showed a markedly longer half-life than MDM2-FL, indicating degradation pathways distinct from the parental protein [53]. The mechanism behind the senescence increase, and behind its loss under cytotoxic stress, remains unresolved. For MDM2-C, overexpression alone likewise induced senescence in human diploid fibroblasts [46].

A related layer of p53-pathway modulation is provided by TP53BP2, whose short isoform 53BP2S lacks the first 123 amino acids of ASPP2-FL (also designated 53BP2L) (Figure 1A) [43]. This N-terminal region binds Ras-GTP at the plasma membrane and stimulates Ras-GTP loading, Raf dimerization and downstream Raf/MEK/ERK signaling. Through this activity, ASPP2-FL (L3) potentiates OIS, whereas 53BP2S, despite retaining the C-terminal p53-binding domain shared by both isoforms, is defective in this Ras/ERK-stimulating activity [54]. Senescence itself, however, was measured with total ASPP2, so the contribution of the ASPP2-FL/53BP2S balance remains untested.

## 6. The CDKN2A Locus and Cyclin D1: Cell-Cycle Regulators Shaped by Alternative Transcript Architecture

The CDKN2A/INK4-ARF locus provides a distinctive example of how alternative first-exon usage combined with alternative splicing generates two structurally unrelated proteins, p16INK4a and ARF (L2), with converging, mutually reinforcing tumor-suppressive functions. Two alternative first exons, E1α and E1β, are each spliced onto common exons 2 and 3, which are read in different frames: transcripts initiated at E1α encode the cyclin-dependent kinase inhibitor p16INK4a, whereas those initiated at E1β encode ARF (Figure 1A, p14ARF in humans, p19ARF in mice), which stabilizes p53 by inhibiting MDM2 [55]. During senescence, p16INK4a accumulates progressively and is used as a biomarker of senescent and aged cells; its loss is a frequent route by which incipient tumor cells bypass the senescence barrier [7,55,56].

The relevance of the ARF arm to senescence is illustrated by a MYC-driven transgenic mouse model of T-cell acute lymphoblastic leukemia, in which MYC inactivation triggers tumor regression that depends in part on cellular senescence. Genetic loss of p19ARF, but not of p53 itself, impaired the induction of senescence, measured by SA-β-gal positivity, p16INK4a induction and senescence-associated histone marks, and blunted macrophage recruitment to the tumor [57]. Because ARF and p16INK4a are translated from transcripts that differ only in first-exon choice and reading frame, changes in the balance of E1α- versus E1β-initiated transcription would be expected to tune both a CDK-inhibitory and a p53-stabilizing arm of the senescence machinery [55], although this has not been tested.

CCND1, encoding the cyclin partner of CDK4/6 that phosphorylates and inactivates RB1 to license the G1/S transition, carries a common germline polymorphism (G870A) that weakens the canonical splice-donor site at the exon 4/intron 4 boundary [67]. The A allele favors retention of intron 4, whose in-frame stop codon causes premature translation termination and produces CCND1b (L3) (Figure 1B), a C-terminally divergent isoform lacking the exon-5-encoded export signal and therefore constitutively nuclear [67,69,70]. CCND1b displays generally greater transforming activity and invasive capacity across multiple tumor types [69,70]. Since cyclin D1-CDK4/6 drives the RB phosphorylation that p16INK4a inhibits to enforce senescence, the CCND1a/CCND1b balance sits immediately upstream of the CDKN2A-RB senescence-execution module. Sustained overexpression of wild-type cyclin D1 (CCND1a) induces cell-cycle arrest and senescence-associated β-galactosidase positivity in primary mouse embryonic fibroblasts [68]. Whether CCND1b opposes this arrest has not been tested, so its contribution to escape remains an inference from its transforming activity and constitutive nuclear localization.

## 7. Nuclear and Telomere-Associated Isoforms

Telomerase reactivation is one of the best-established routes by which cancer cells escape replicative senescence [91]. Several hTERT splice variants, commonly termed the α-, β- and γ-deletion transcripts (L2), remove different portions of the reverse transcriptase domain and are detected across normal and malignant tissues [74,92]. The α-deletion arises from a cryptic 3′ splice acceptor in exon 6; the β-deletion results from skipping of exons 7 and 8, which shifts the reading frame and introduces a premature stop codon; and the γ-deletion arises from removal of an in-frame segment within exon 11 (Figure 1B) [74]. The α- and γ-deletions remain in frame and act as dominant negatives, whereas β-deletion transcripts are largely degraded by nonsense-mediated decay [74]. In lung carcinoma, the constituent ratios of the deletion transcripts, most strongly that of the β-deletion, were inversely correlated with telomerase activity, whereas total hTERT transcript abundance was not [73]. In colorectal cancer, telomerase activity tracked the balance between the full-length and β variants, and the percentage of full-length transcript was an independent prognostic factor for survival [72]. hTERT alternative splicing also operates physiologically, as shown by a shift in hTERT splicing in target lymphocytes exposed to regulatory T cells [93]. Together, these observations support hTERT alternative splicing as a potential regulator of telomerase activity and senescence escape, although it remains unresolved whether experimentally altering the hTERT isoform ratio is sufficient to modulate senescence.

A different route to senescence engages nuclear architecture directly, independently of telomere length. LMNA generates prelamin A and, through activation of a cryptic splice donor in exon 11 (Figure 2A), the senescence-relevant isoform, progerin (L1) [94,95]. The resulting 50-amino-acid deletion removes the ZMPSTE24 processing site, leaving progerin permanently farnesylated and stably anchored in the nuclear envelope. The same cryptic splice site is used at much lower levels in normal aged cells, and its inhibition can reverse age-associated nuclear defects [66]. Progerin accumulation triggers senescence and oxidative damage in human coronary endothelial cells [63], and it is associated with cardiomyocyte dysfunction in dilated cardiomyopathy [65]. Altered LMNA splice-variant expression has also been reported across several carcinomas [96]. An engineered lamin A mutant with defective ZMPSTE24 processing and a non-phosphorylatable CDK1 site produces a p53-independent senescence-like arrest and reduced tumorigenicity in vivo [97]. However, this mechanism has not been demonstrated for authentic splicing-generated progerin. LMNA also produces lamin C (L4), through a distinct event, in which the transcript retains the proximal part of intron 10 and uses its own polyadenylation site, never reaching exon 11 [85]. Lamin C therefore lacks the CaaX motif required for farnesylation and is not a substrate for the progerin-generating event [64]; whether it modulates senescence or proliferation independently remains untested.

PML provides a related example in which alternative splicing generates isoforms that assemble into PML nuclear bodies (PML-NBs) of isoform-specific composition, which are well established as organizing centers for p53 activation. Of the seven known PML isoforms, only PML-IV (L1) has been shown to induce premature senescence [75]. PML-IV contains a conserved 13-amino-acid C-terminal extension, generated by a distal 5′ splice donor site in exon 8 that reads in-frame into intron 8 before a premature stop codon, a sequence termed exon 8b in the PML literature (Figure 2B). This extension mediates direct binding to the ARF tumor suppressor, enhancing p53 SUMO-1 conjugation and activation, thereby inducing senescence [76]. PML-NB size and number also increase during replicative senescence in primary human fibroblasts [98], but this remodeling has not been attributed to any specific isoform.

## 8. Context-Dependent Isoform Regulation of Senescence in Cancer

The isoform switches that follow do not belong to the gene groups considered so far and are grouped instead by the strength of their link to senescence.

### 8.1. Isoform Switches with a Documented Senescence Phenotype

Androgen deprivation therapy (ADT) can induce androgen-deprivation-induced senescence (AIS) in prostate cancer [71]. Resistance to ADT and progression to castration-resistant prostate cancer are strongly associated with androgen receptor (AR) variant 7 (AR-V7) (L1), a constitutively nuclear, ligand-independent AR isoform generated by inclusion of a cryptic exon that truncates the ligand-binding domain (Figure 1B) [99,100]. In prostate cancer models, AR-V7 promotes escape from AIS through SKP2 activation, which drives degradation of the CDK inhibitor p27 and cell-cycle re-entry. SKP2 is suppressed by ADT but reactivated during tumor progression, and its inhibition restrains androgen-independent growth and enhances ADT efficacy, supporting a role for the AR-V7-SKP2-p27 axis in senescence escape [71].

The balance between senescence and apoptosis is also influenced by BCL2-family proteins, several of which are alternatively spliced. Senescent cells generally show high levels of the anti-apoptotic proteins Bcl-2, Bcl-xL and Mcl-1 [81], and in one system, tetraploidization-induced senescence, combined loss of BAX and BAK favors senescence over apoptosis in tetraploid cells [101]. MCL1 generates the pro-apoptotic MCL-1S isoform (L3) through exon 2 skipping, which replaces the C-terminal BH1/BH2/TM-containing region of MCL-1L with an unrelated tail [77,78] (Figure 2B). However, Mcl-1’s anti-senescence activity maps to an internal loop shared by both isoforms [79]. Senescent tumor cells depend on Mcl-1 for survival, but that dependence has been demonstrated only for the total protein [80]. BCL2L1 (L3) instead uses alternative 5′ splice-site selection within exon 2: the proximal site produces the long, anti-apoptotic Bcl-xL and the distal site produces the shorter, pro-apoptotic Bcl-xS, which lacks the BH1 and BH2 domains [37] (Figure 2B). Whether the senescence-associated increase in Bcl-xL and Mcl-1 reflects altered isoform ratios or total protein induction remains unresolved, although shifting the BCL2-family balance toward apoptosis can enhance elimination of senescence-prone cancer cells [81]. Bcl-xL itself has context-dependent effects: its overexpression suppresses senescence entry in low-grade pancreatic lesions but inhibits apoptosis in high-grade lesions, whereas genetic loss induces senescence [82].

Isoform switching also regulates the SASP, as shown for EXOC7, a subunit of the exocyst complex that tethers secretory vesicles to the plasma membrane [102]. An unbiased screen identified PTBP1 as a driver of exon 7 skipping in EXOC7, favoring the short EXOC7-S isoform (L1) over the EXOC7-L isoform (Figure 2C). EXOC7-S showed increased ERK1/2-mediated phosphorylation and a distinct subcellular localization relative to EXOC7-L, consistent with enhanced exocyst activity and secretion. Overexpression of EXOC7-S produced a higher SASP than EXOC7-L and partially restored IL-6 and IL-8 production after PTBP1 depletion. In vivo, EXOC7 knockdown attenuated the SASP and immune-cell infiltration in an oncogenic-RAS-driven mouse model without affecting the underlying senescent growth arrest, phenocopying PTBP1 loss [24].

### 8.2. Isoform Switches with an Unresolved Link to the Senescence Phenotype

Four further pairs involve proteins central to the senescence program but lack an isoform-resolved senescence readout, each for a different reason: the senescence evidence comes from a different allele, from a different endpoint, from biochemistry alone, or from a clinical association with no senescence readout (Table 2). Cancer cells with BRCA1 exon 11 mutations predominantly express BRCA1-Δ11q (L4), which confers resistance to PARP inhibitors, a phenotype with no reported connection to senescence [59]. The senescence and premature aging described in mice carrying a complete deletion of exon 11 arise from an engineered allele [58], not from the naturally spliced isoform. Retention of the intron separating the two coding exons of CDKN1A produces p21L (L4), a cytoplasmic isoform lacking the C-terminal cyclin- and PCNA-binding motifs of p21C [61]. Whether it retains anti-proliferative activity is disputed [60,61], and it has been characterized only for proliferation and cell-cycle arrest. In-frame skipping of exon 8 generates SIRT1-ΔExon8 (L3), a variant that retains weak deacetylase activity and forms a feedback loop with p53 [62]; its contribution to senescence has not been tested. STAT3 is spliced into the active STAT3α and the truncated STAT3β (L4) [84], which has partly STAT3α-independent functions [103]. A higher STAT3β-to-STAT3α ratio is associated with longer survival in AML, but no senescence phenotype has been reported for the isoform itself [83]. In each case, the open question is not whether the isoform is biologically active, but whether its effects extend to cellular senescence.

## 9. Therapeutic Targeting of Senescence-Relevant Splicing Events

For several senescence-relevant genes, functional output depends on the relative abundance of alternative isoforms rather than on an irreversible genetic alteration, creating a potential pharmacological opportunity to modify the isoform balance or its downstream consequences. Therapeutic strategies can aim to induce senescence in tumor cells that have not yet arrested, prevent escape from an established senescent state, or eliminate or functionally neutralize senescent cells once formed.

Splice-switching antisense oligonucleotides (ASOs), which redirect spliceosome choice at a target pre-mRNA without altering the underlying DNA sequence, have been used experimentally to shift the balance toward the pro-apoptotic short isoforms of both BCL2L1 and MCL1. This approach is more closely aligned with eliminating vulnerable senescent cells than with inducing or maintaining senescence, as it aims to restore apoptotic sensitivity in tumors that depend on the long, pro-survival isoform [34,37]. Small-molecule spliceosome modulators, such as the SF3B1-targeting agents herboxidiene and pladienolide B, provide a complementary strategy: in the specific cellular contexts tested, global perturbation of spliceosome activity induced senescence through the MDM4 splicing switch, although this approach is pharmacologically non-specific and affects the splicing of many genes simultaneously [21,23].

For senescence escape, corrective strategies can target either the splicing event itself or an immediate downstream effector. The CCND1a/CCND1b switch represents a potential example of the former [70], although its effect on senescence remains experimentally untested. More directly, inhibition of the AR-V7 downstream effector SKP2 counteracts AR-V7-driven p27 degradation and limits androgen-independent growth, supporting this pathway as a potential target for restricting escape from therapy-induced senescence [71].

The PTBP1-EXOC7 splicing axis illustrates a conceptually distinct strategy, suppressing the harmful consequences of persistent senescence while retaining the growth-arrested state itself. This has so far been demonstrated by knockdown and by splice-switching ASOs in cultured cells, rather than tested as a therapeutic intervention [24], but could be particularly relevant when senescence itself remains desirable and the SASP becomes the primary source of tumor-promoting inflammation.

The MDM2 data show that a splicing-based intervention promoting senescence under basal conditions can lose its effect under cytotoxic stress, and splice-modulating therapies may therefore need to be deliberately sequenced or combined with cytotoxic or targeted agents rather than assumed to produce additive effects [53]. None of these strategies has reached clinical evaluation for a senescence-relevant isoform, and none has yet combined a splicing-specific intervention, a clinically relevant tumor model and a realistic treatment schedule in a single study.

## 10. Limitations of the Current Evidence Base and Open Questions

The evidence linking alternative isoforms to cellular senescence remains highly uneven across genes and biological contexts. The TP53 family and the MDM4-spliceosome axis are supported by multiple in vitro and in vivo studies with defined molecular mechanisms [23,39,40,41], whereas MDM2, CCND1 and PML rest on a narrower experimental base [53,68,75], ranging from single-study observations to mechanistically incomplete associations.

Several cancer-relevant splicing programs also lack direct evidence for a role in senescence. These include CDKN1A and the BCL2L1 splicing program when considered independently of its downstream effects on apoptotic balance [37,60,61]. The same applies to STAT3β, whose relationship to senescence remains untested despite a well-established anti-leukemic function [83].

Many of these mechanisms have been demonstrated in a limited range of cell types or tumor contexts, such as T lymphocytes for Δ133p53/p53β, MEFs for ΔNp73, MCF-7 breast cancer cells for MDM2 variants, AML for STAT3β and prostate cancer for AR-V7 [38,41,53,71,83]. Whether these relationships are conserved across tissue types, or how they interact when multiple isoform switches occur simultaneously within the same tumor, remains largely unexplored.

The PTBP1-EXOC7 findings connect splicing to the secretory component of senescence, and the p19ARF results link a product of an alternatively initiated locus to its immune component [24,57]. It remains unclear, however, how isoform expression varies among individual senescent cell subpopulations. Resolving this heterogeneity will be important because senescent cells within the same tumor may differ substantially in their persistence, secretory activity, immune interactions and susceptibility to elimination.

The relationship between isoform-level regulation and cellular plasticity is supported even less directly. The available evidence connects two separately characterized phenomena: an isoform switch that affects a senescence-relevant function and the association of the resulting cellular state with plasticity. The proposed sequence, from an isoform switch to plasticity, is therefore supported only as far as the senescence step.

## 11. Future Perspectives

Moving beyond associative observations will require isoform-resolved measurement in senescent cells. Single-cell and spatial transcriptomics, with long-read sequencing to resolve full-length isoforms directly rather than infer them from short-read junction counts, would show whether an isoform switch is uniform across a senescent population or restricted to particular subpopulations. Mapping isoform ratios across entry, persistence and escape within one system would test whether they change with senescence stage rather than marking a static difference between senescent and proliferating cells. Two relationships are currently classified as senescence-associated, which reflects how rarely such ratios have been measured rather than how rarely they change.

For relationships graded as senescence-associated or mechanistically adjacent, splice-switching experiments that shift isoform ratios with ASOs or minigene systems offer the most direct route to establishing or refuting a causal role. Several of these mechanisms were characterized in isolated cell populations, so three-dimensional and in vivo models that incorporate tumor, stromal and immune-cell heterogeneity will be needed to test whether the relationships hold in a realistic tumor context. It also remains to be shown whether an isoform ratio can be shifted in vivo selectively enough to modify senescence without the global effects that accompany spliceosome inhibition.

Nuclear architecture shapes splicing outcome in general, and CTCF clustering on nuclear speckles is required to establish the senescence-associated splicing program, but neither has yet been connected to any individual isoform switch. Whether the switches that govern commitment, persistence, escape and SASP composition are set by that layer is unresolved.

The step that would change the field most is also the one taken least often. Measuring an isoform ratio and a senescence outcome in the same system, over time, with the ratio manipulated rather than only observed, is what separates a demonstrated mechanism from an association.

## 12. Conclusions

Alternative splicing and closely related mechanisms, including alternative promoter usage and alternative reading-frame selection, can modulate distinct outputs of the senescence program, such as commitment, persistence, escape and SASP composition, rather than acting as uniform determinants of whether senescence occurs.

Where direct experimental evidence is available, isoform regulation provides a plausible molecular mechanism through which a single locus can generate functionally divergent effects on tumor suppression and senescence. The available findings also suggest potential connections between isoform regulation, cellular plasticity, EMT-associated and stemness programs, and the senescent immune microenvironment. From a therapeutic perspective, splice-switching and spliceosome-targeted approaches provide potential non-mutational strategies for modifying senescence or its downstream consequences, although none has yet reached clinical evaluation. Alternative splicing and related transcript-level mechanisms therefore represent a mechanistically plausible but still substantially underexplored dimension of cancer-associated senescence.

Several of the isoform pairs reviewed here show that alternative splicing, promoter usage or reading-frame selection does not merely generate structurally distinct protein variants, but variants with directly opposing functional consequences, as in MDM4-FL versus MDM4-S, Bcl-xL versus Bcl-xS, ΔNp63 versus TAp63, and EXOC7-L versus EXOC7-S. The term “isoform” does not by itself convey this distinction, since it denotes any protein variant arising from a single gene, regardless of whether the variants act similarly, independently or in direct functional opposition to one another. Where such antagonism has been directly demonstrated, it is worth stating explicitly rather than leaving it implicit under that shared label. In most other cases, the functional relationship between the variants has not yet been resolved.

## Figures and Tables

**Figure 1:**
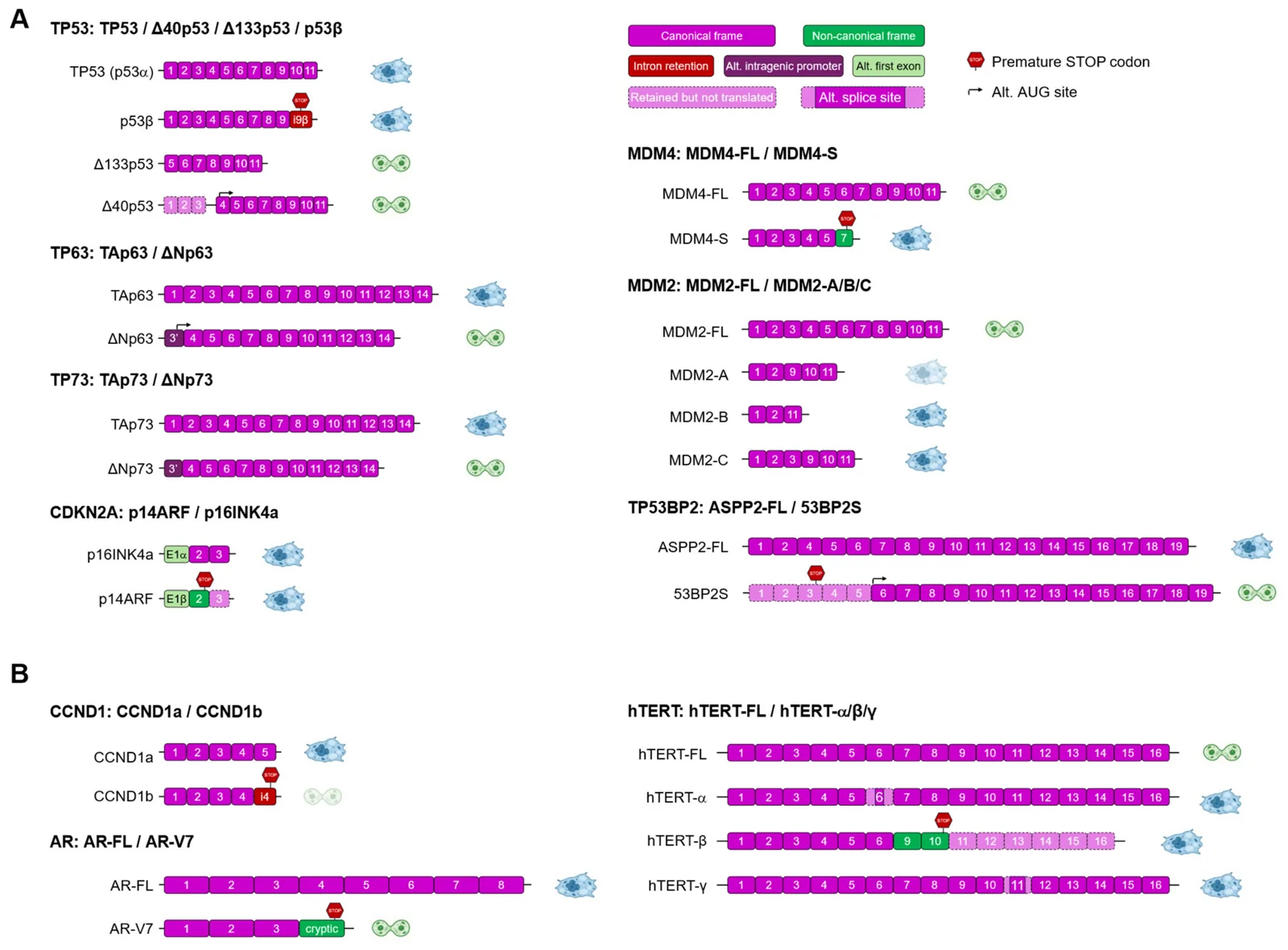
Isoform architectures of genes acting on senescence entry and escape. Isoform pairs and groups are organized by the functional axis they most directly affect: (**A**) senescence entry or commitment; (**B**) senescence escape. Boxes represent exons numbered according to the MANE Select transcript of each gene (TP53BP2 is numbered to include the cassette exon 3); only exons present in the mature transcript of each isoform are drawn, and boxes labeled “i” followed by a number denote intronic sequence present in the mature transcript, whether retained in full or included through use of an alternative splice site. Color and symbol codes are given in the key. Icons to the right of each isoform indicate the senescence-related outcome supported by the available evidence: joined green cells denote cell-cycle progression or senescence escape, and the flattened blue-gray cell denotes senescence entry, persistence or survival. Both icons are shown where an isoform has context-dependent effects, a faded icon indicates an outcome inferred but not directly tested, or tested without reaching significance, as for MDM2-A; and no icon is shown where no specific outcome has been assigned. For Δ40p53, only the alternative-initiation route is drawn; this isoform is also generated by retention of intron 2. For MDM4-S, the icon denotes senescent-cell survival rather than senescence entry. For AR-V7, “cryptic” denotes an exon absent from the canonical transcript, generated by inclusion of an intronic sequence.

**Figure 2:**
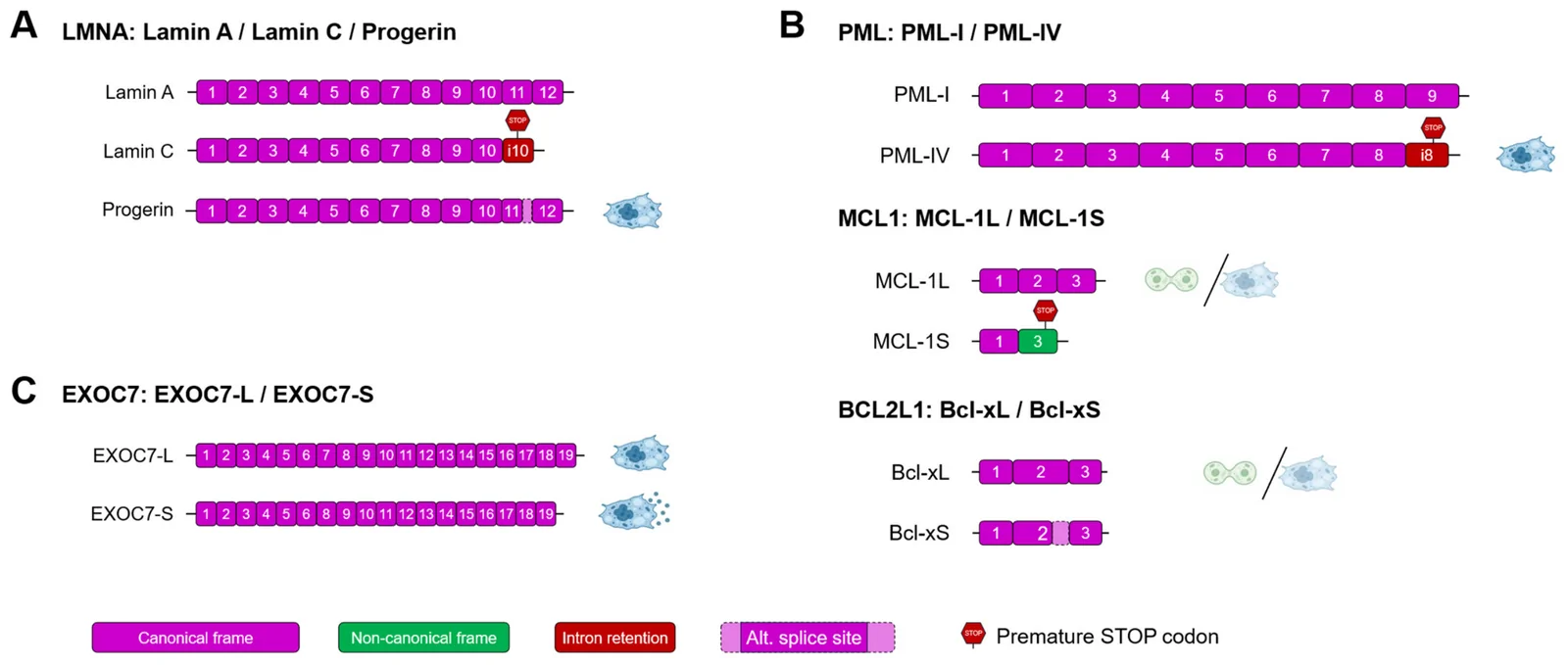
Isoform architectures of genes acting on senescence entry through nuclear integrity, senescent-cell persistence and SASP regulation: (**A**) senescence entry through loss of nuclear integrity (LMNA); (**B**) senescent-cell persistence or survival (PML, MCL1, BCL2L1); (**C**) SASP regulation (EXOC7). Exon numbering, color and symbol coding and outcome icons are as in Figure 1. Dots surrounding the senescent-cell icon indicate SASP secretion and are shown only for EXOC7-S. The lamin C transcript reads into intron 10 (i10) to its own stop codon and polyadenylation site and does not include exons 11 and 12.

**Table 1:** Evidence level assigned to each isoform pair, grouped by the functional axis of the senescence program it most directly affects.

Functional Axis	Gene/Product(s)	Evidence Level	Section	Reference(s)
Senescence entry/commitment	Δ133p53/p53β (TP53)	**L1—Direct**	4.1	[39,41,45]
	Δ40p53/p53α (TP53)	**L1—Direct** *(* *Context-dependent: antagonizes p53-dependent growth suppression in p53-null lung cancer cells; induces senescence in hepatocellular carcinoma)*	4.1	[51,52]
	ΔNp73/TAp73 (TP73)	**L1—Direct**	4.2	[38]
	ΔNp63/TAp63 (TP63)	**L1—Direct**	4.2	[40]
	MDM2-A/-B/-C (MDM2)	**L1—Direct** *(* *Confined to basal conditions; lost under cytotoxic stress)*	5	[53]
	MDM4-FL/MDM4-S (MDM4)	**L1—Direct** *(Attributable to loss of MDM4-FL; MDM4-S overexpression did not affect senescence onset)*	5	[23]
	ASPP2-FL/53BP2S (TP53BP2)	**L3—Mechanistically adjacent** *(Isoform difference shown for Ras/ERK activation; senescence measured with total ASPP2)*	5	[43,54]
	p16INK4a/ARF (CDKN2A)	**L2—Senescence-associated** *(* *Both products individually have direct evidence for senescence; the E1α/E1β choice itself has not been manipulated)*	6	[7,55,56,57]
	BRCA1-FL/BRCA1-Δ11q (BRCA1)	**L4—Unresolved** *(Isoform-specific senescence* *untested in human cells; mouse evidence from an engineered allele)*	8.2	[58,59]
	p21C/p21L (CDKN1A)	**L4—Unresolved** *(Characterized only for cell-cycle arrest, with conflicting results between studies)*	8.2	[60,61]
	SIRT1/SIRT1-ΔExon8 (SIRT1)	**L3—Mechanistically adjacent** *(Retains weak deacetylase activity and forms a feedback loop with p53; contribution to senescence untested)*	8.2	[62]
	Lamin A/progerin (LMNA)	**L1—Direct** *(Progerin expression and splice blocking in human cells; the engineered lamin A mutant mechanism is not demonstrated for splicing-generated progerin)*	7	[63,64,65,66]
	Lamin A/lamin C (LMNA)	**L4—Unresolved** *(Senescence link untested)*	7	[64]
Senescence escape	CCND1a/CCND1b (CCND1)	**L3—Mechanistically adjacent** *(CCND1a overexpression induces senescence; the contribution of CCND1b to escape is inferred, not tested)*	6	[67,68,69,70]
	AR-FL/AR-V7 (AR)	**L1—Direct**	8.1	[71]
	hTERT full-length/α, β, γ deletions (TERT)	**L2—Senescence-associated *(*** *Correlative)*	7	[72,73,74]
Senescent-cell persistence/survival	PML-IV/Other PML isoforms (PML)	**L1—Direct** *(Specific to PML-IV; PML-NB remodeling is correlative and not isoform-specific)*	7	[75,76]
	MCL-1L/MCL-1S (MCL1)	**L3—Mechanistically adjacent** *(Senescent cells depend on Mcl-1, but the dependence is not isoform-resolved; the antisenescence domain lies in the region shared by both isoforms)*	8.1	[77,78,79,80]
	Bcl-xL/Bcl-xS (BCL2L1)	**L3—Mechanistically adjacent** *(Bcl-xL loss or overexpression alters senescence at the protein level; the xL/xS switch itself is untested in senescence)*	8.1	[37,81,82]
SASP regulation	EXOC7-L/EXOC7-S (EXOC7)	**L1—Direct** *(Gain- and loss-of-function* in vitro *and* in vivo*)*	8.1	[24]
	STAT3α/STAT3β (STAT3)	**L4—Unresolved** *(S* *enescence link untested)*	8.2	[83,84]

Direct (L1) evidence is available for nine genes: TP53, TP63, TP73, MDM2, MDM4, LMNA, AR, PML and EXOC7; TP53 contributes two of the ten L1 pairs. Of the remaining eleven pairs, two are graded L2, five L3 and four L4. References list the studies supporting the assigned evidence level. In the Evidence Level column, the assigned level is given in bold and any qualifying note in italics; cell shading encodes the same level (green, L1; yellow, L2; blue, L3; grey, L4).

**Table 2:** Experimental model, species, senescence trigger, isoform-generating mechanism and type of molecular validation for each isoform pair.

Isoform Pair (Gene)	Experimental Model	Species	Senescence Trigger	Isoform-Generating Mechanism	Type of Molecular Validation	Ref.
Δ133p53/p53β (TP53)	Human fibroblasts; CD8+ T lymphocytes (blood and tumor-infiltrating)	Human	Replicative (not oncogene-induced)	Internal promoter (intron 4); alt. 3′ splice site (intron 9)	Isoform-specific knockdown and overexpression; SA-β-gal, p21, replicative lifespan	[39,41]
Δ40p53/p53α (TP53)	Hepatocellular carcinoma lines (HuH-1, HepG2, Hep3B, HuH-7, PLC/PRF/5); H358 lung cancer cells (colony formation); mouse fibroblasts (transactivation)	Human; mouse	None; senescence induced by Δ40p53 expression itself	Intron 2 retention + alt. translation initiation	Targeted endogenous allele; TP53 knockdown and rescue; ectopic expression; SA-β-gal, p21	[51,52]
ΔNp73/TAp73 (TP73)	Primary MEFs; nude mice	Mouse	Replicative; oncogenic RAS	Alternative promoter	Isoform expression; immortalization frequency, rescue of RAS-induced senescence, tumor formation	[38]
ΔNp63/TAp63 (TP63)	Tet-on inducible mouse; MEFs	Mouse	Induced TAp63 expression	Alternative promoter	Inducible isoform expression in vivo; senescence program, tumor suppression	[40]
MDM2-A/-B/-C (MDM2)	MCF-7 cells; human diploid fibroblasts	Human	None (unstressed); doxorubicin; SRSF7 downregulation (fibroblasts)	Exon skipping	Overexpression of each variant; SA-β-gal-positive fraction	[46,53]
MDM4-FL/MDM4-S (MDM4)	Primary human fibroblasts (BJ, IMR-90, WI-38); HCT116 colon cancer cells	Human	Replicative; oxidative stress (H_2_O_2_); telomere uncapping; spliceosome inhibition	Exon 6 skipping, frameshift, PTC	MDM4-FL-specific shRNA: SA-β-gal, p21, proliferation; spliceosome inhibition reproduces the shift; MDM4-S overexpression affects survival, not onset	[23]
ASPP2-FL/53BP2S (TP53BP2)	293T (isoform comparison); human fibroblasts and keratinocytes (senescence); ASPP2+/−;p53+/− MEFs	Human; mouse	H-RasV12	Cassette-exon (exon 3) inclusion + translational reinitiation	ASPP2-FL vs. 53BP2S for Ras-GTP binding and ERK activation; senescence measured with total ASPP2	[43,54]
p16INK4a/ARF (CDKN2A)	MYC-driven mouse T-ALL; human ALL cohort	Mouse; human	MYC inactivation	Alt. first-exon usage + alt. reading frame	Genetic loss of p19ARF, not of the E1α/E1β choice; SA-β-gal, p16INK4a	[57]
BRCA1-FL/BRCA1-Δ11q (BRCA1)	Mouse with complete exon 11 deletion; human tumors and cells with exon 11 mutations	Mouse; human	Loss of full-length Brca1; none for Δ11q	Exon 11 skipping (in-frame)	Mouse genetics, SA-β-gal, premature aging; isoform expression with PARPi and cisplatin response	[58,59]
p21C/p21L (CDKN1A)	A375 and HCT-116 cells; B-cell leukemia/lymphoma lines and primary CLL samples	Human	None (CDK11 or SF3B1 inhibition)	Intron retention (novel C-terminus)	Inducible expression of each isoform, mass spectrometry, localization, proliferation and pRB phosphorylation; no senescence endpoint	[60,61]
SIRT1/SIRT1-ΔExon8 (SIRT1)	16 human epithelial lines, normal and cancer-derived; mouse lines and tissues	Human; mouse	None; isoforms quantified after stress	Exon 8 skipping (in-frame)	Isoform-specific qRT-PCR; deacetylase activity, p53 acetylation; no senescence endpoint	[62]
Lamin A/progerin (LMNA)	Human coronary endothelial cells; aged human cells	Human	Progerin accumulation (ectopic and physiological)	Cryptic 5′ donor site within exon 11	Isoform expression, SA-β-gal, DNA damage; splice blocking, farnesylation inhibition	[63,66]
Lamin A/lamin C (LMNA)	Not assessed for senescence	—	—	Intron 10 retention + alt. polyadenylation	None	[64,85]
CCND1a/CCND1b (CCND1)	Primary MEFs (CCND1a only)	Mouse	Sustained cyclin D1 overexpression	Intron 4 retention (G870A)	Overexpression, SA-β-gal; CCND1b not tested	[68]
AR-FL/AR-V7 (AR)	LNCaP, LNCaP-AI, C4-2, PC-3 and 22Rv1 cells; public human datasets	Human	Androgen deprivation	Cryptic exon inclusion, PTC	Isoform knockdown and expression, SKP2–p27 axis, SA-β-gal, androgen-independent growth	[71]
hTERT full-length/α, β, γ deletions (TERT)	Lung carcinoma and colorectal cancer specimens	Human	None (correlative)	Cryptic 3′ acceptor (α); exon 7–8 skipping (β); internal deletion in exon 11 (γ)	Isoform-specific RT-PCR against telomerase activity; no manipulation	[72,73]
PML- IV/Other PML isoforms (PML)	Primary fibroblasts	Mouse and human	PML-IV overexpression	Distal 5′ donor site in exon 8, reading into intron 8	Isoform-specific overexpression; SA-β-gal, p53 SUMOylation	[75,76]
MCL-1L/MCL-1S (MCL1)	HCT116 and head-and-neck carcinoma lines; HCT116 xenografts; senescent prostate tumor cells in Pten-null mice	Human; mouse	Doxorubicin (chemotherapy-induced); therapy-induced in vivo	Exon 2 skipping, frameshift	Dependence on total Mcl-1, loop-domain mapping; isoform ratio not manipulated	[79,80]
Bcl-xL/Bcl-xS (BCL2L1)	Pancreas-specific KrasG12D mice; human PanIN/PDAC	Mouse; human	Oncogene-induced (KRAS)	Alt. 5′ splice site within exon 2	Total Bcl-xL overexpression and knockdown, isoform ratio not manipulated	[82]
EXOC7-L/EXOC7-S (EXOC7)	IMR90 ER:RAS; oncogenic RAS mouse model	Human; mouse	Oncogene-induced (4-OHT)	Exon 7 skipping	siRNA screen, overexpression of both isoforms, knockdown, IL-6/IL-8, SASP and immune infiltration in vivo	[24]
STAT3α/STAT3β (STAT3)	AML patients; transgenic Stat3β mice (PTEN, MLL-AF9)	Human; mouse	None (senescence not assessed)	Alt. 3′ splice site (C-terminal truncation)	Isoform ratio against survival; transgenic balanced expression; no senescence endpoint	[83]

Where an isoform pair has been characterized in more than one system, each system is listed. “No senescence endpoint” indicates that the isoform was characterized without measuring a senescence phenotype. Entries stating that the isoform ratio was not manipulated indicate that the senescence readout was obtained for the total gene product. References list the studies describing the experimental systems shown. Abbreviations: ALL, acute lymphoblastic leukemia; CLL, chronic lymphocytic leukemia; PanIN, pancreatic intraepithelial neoplasia; PARPi, PARP inhibitor; PDAC, pancreatic ductal adenocarcinoma; PTC, premature termination codon; qRT-PCR, quantitative reverse-transcription PCR.

## Data Availability

No new data were created or analyzed in this study. Data sharing is not applicable to this article.

## Abbreviations

The following abbreviations are used in this manuscript:

ADT	Androgen deprivation therapy
AIS	Androgen-deprivation-induced senescence
AML	Acute myeloid leukemia
AR	Androgen receptor
ASO(s)	Antisense oligonucleotide(s)
EMT	Epithelial-mesenchymal transition
MEFs	Mouse embryonic fibroblasts
OIS	Oncogene-induced senescence
PARP	Poly(ADP-ribose) polymerase
PML-NB(s)	PML nuclear body/bodies
SASP	Senescence-associated secretory phenotype
SA-β-gal	Senescence-associated β-galactosidase
T-ALL	T-cell acute lymphoblastic leukemia
